# Assessing Potentially Inappropriate Prescribing in Community-Dwelling Older Patients Using the Updated Version of STOPP-START Criteria: A Comparison of Profiles and Prevalences with Respect to the Original Version

**DOI:** 10.1371/journal.pone.0167586

**Published:** 2016-12-01

**Authors:** Encarnación Blanco-Reina, Maria Rosa García-Merino, Ricardo Ocaña-Riola, Lorena Aguilar-Cano, Jennifer Valdellós, Inmaculada Bellido-Estévez, Gabriel Ariza-Zafra

**Affiliations:** 1 Pharmacology and Therapeutics Department, Medical School, Málaga Biomedical Institute (IBIMA), University of Málaga, Málaga, Spain; 2 Health District of Córdoba Sur, Córdoba, Spain; 3 Statistics Department, Andalusian School of Public Health, Granada, Spain; 4 Health District of Málaga, Málaga, Spain; 5 Health Area of Málaga Norte, Málaga, Spain; 6 Geriatrics Department, University Hospital of Albacete, Albacete, Spain; Boston University, UNITED STATES

## Abstract

Emerging and changing evidence made it necessary to update STOPP-START criteria, and version 2 was published recently. In this study the objectives were to determine the prevalence of potentially inappropriate medication prescribing (PIM) in primary care using STOPP versions 1 (v1) and 2 (v2), as well as 2012 AGS Beers criteria, and analyze the factors associated with inappropriate prescribing according to STOPP/START v2. A cross-sectional study was carried out including community-dwelling older adults over the age of 65. Sociodemographic, clinical, functional and comprehensive drug therapy data were collected. The primary endpoint was the percentage of patients receiving at least one PIM. This variable was measured using three tools: STOPP v1, 2012 AGS Beers criteria and STOPP v2. Similarly, the percentage of patients receiving at least one potential prescribing omission (PPO) was calculated using START versions 1 and 2. A total of 1,615 prescriptions were reviewed. The median number of medications per patient was 7.1 drugs (±3.8). The prevalence of elderly people exposed to polypharmacy (≥5 medications) was 72.9%, whereas 28.4% of the participants took ≥10 drugs regularly. PIM were present in 18.7%, 37.3% and 40.4% of participants, according to the STOPP v1, 2012 Beers criteria and STOPP v2, respectively. According to STOPP v2, the number of medications taken (OR: 1.14, 1.06–1.25), the presence of a psychological disorder (OR: 2.22, 1.13–4.37) and insomnia (OR: 3.35, 1.80–6.32) were risk factors for taking a PIM. The prevalence of PPOs was 34.7% and 21.8% according to version 1 and 2, respectively. In conclusion, STOPP-START criteria have been remarkably modified, which is evidenced by the different prevalence rates detected using version 2, as compared to version 1. In fact, the level of agreement between version 1 and the updated version is only moderate. Special attention should be paid on benzodiazepines, which keep being the most frequent PIM.

## Introduction

Inappropriate medication prescribing in older adults has become a public health concern due to its high prevalence, associated negative outcomes, and increased costs [1, 2]. Exposure to inappropriate medications is a major risk factor for adverse drug events (ADEs) [3–5], and it has been linked to increased morbidity, hospitalization and mortality rates. A number of strategic approaches have been developed to optimize medication use among older people, including the use of screening tools for the detection of potentially inappropriate medication prescribing. The USA Beers Criteria and the European Screening Tool of Older Person’s Potentially Inappropriate Prescriptions (STOPP) and Screening Tool to Alert doctors to the Right Treatment (START) are the most widely used criteria for the detection of prescription errors [6]. In the past, the studies using STOPP consistently detected a higher prevalence of potentially inappropriate medication (PIMs) prescribing in the European population than the studies using Beers criteria [7, 8]. In contrast, the updated 2012 AGS Beers criteria [9]–which provided a more-dynamic list that is more in line with clinical practice–has been demonstrated to have higher sensitivity than the original STOPP criteria [10].

Although both tools are useful for medical prescribers, the STOPP/START tool has been proven to be somewhat superior in detecting and preventing ADEs [3, 11]. Additionally, a recent systematic review and meta-analysis of randomized controlled studies with STOPP/START criteria has found evidence that the use of STOPP/START reduces falls, delirium episodes, hospital length-of-stay, care visits and medication costs [12], which proves its effectiveness in clinical practice.

Notwithstanding the above, the STOPP/START tools should be continuously updated to incorporate emerging or even changing evidence and new drugs approved. In the updated STOPP/START version, outdated and less-relevant criteria have been removed and new evidence-based items have been incorporated. In fact, the STOPP/START version 2 shows a 31% increase in the number of indications with respect to version 1 [13]. Most new items are based on evidence provided by clinical trials and recommendations from European panels of experts.

No research using the updated version of STOPP/START on inappropriate prescribing in the European Union has been conducted so far. It would therefore be interesting to investigate if the updated version yields different prevalence rates and improves the applicability of STOPP/START. Thus, the purpose of this study was to determine the prevalence of PIMs in primary care using STOPP versions 1 (v1) and 2 (v2), as well as 2012 AGS Beers criteria. Additional objectives included: (i) to determine the rate of potential prescribing omissions (PPOs) according both, to v1 and the updated version of START; (ii) to compare the specificity and sensitivity of the tools used by calculating the level of agreement among criteria; and (ii) to analyze the factors associated with inappropriate prescribing according to STOPP/START v2.

## Material and Methods

The authors declare that this clinical research was devised in line with the ethical standards laid down in the Helsinki Declaration (Fortaleza 2013) and that special attention was paid to ensuring informed consent from all patients prior to their inclusion as well as to the confidentiality of all personal data. This study was classified as a post-authorization epidemiological study by the Spanish Medications & Healthcare Products Agency. The study was approved by the Clinical Research Ethics Committee Regional of Málaga.

### Study Design, Setting and Subjects

This was a cross-sectional study. The study population included community-dwelling residents over the age of 65 living in Málaga, Spain. Patients were recruited from four primary care centers using stratified random sampling to select a representative sample of the population, with proportional allocation of the population to the size of each healthcare center. Patients aged 65 and older who were living in the community and provided written informed consent to take part in the study were included. Considering an overall prevalence of inappropriate prescribing in primary care in Europe of p = 22% [14], a confidence level of 1-α = 0.95, an absolute accuracy of 5.4% and a design effect δ = 1.0, we obtained a sample size of 225 patients. Patients were randomly selected in each healthcare center from a general list of healthcare cards issued by the National Health System. Data were collected during 2015.

### Data Collection

Data were primarily collected through interviews with participants by using a structured questionnaire complemented with a revision of medication packaging and medical records. A variety of sociodemographic, clinical and functional data were obtained. The Charlson Comorbidity Index (CCI), Katz Index of Independence in Activities of Daily Living, and Lawton Instrumental Activities of Daily Living Scale were calculated for each patient. Cognitive function was evaluated using the Short Portable Mental Status Questionnaire, and mood status was assessed using the Geriatric Depression Scale (GDS-15). Full data on dosage and duration of drug treatment were compiled. Each drug was assigned the corresponding Anatomical Therapeutic Chemical classification code. All interviews were performed by three of the authors, who are all specialized in Family Medicine.

### Statistical Analysis

The primary endpoint was the percentage of patients receiving at least one PIM. This concept was measured using three tools: STOPP v1, 2012 AGS Beers criteria and STOPP v2. Similarly, the percentage of patients receiving at least one PPO was calculated using START versions 1 and 2.

Exploratory Data Analysis and frequency tables were used to describe all variables. Multivariate Logistic Regression was used to examine factors related to PIMs and PPOs (according to the updated STOPP/START). To ensure goodness of fit, further diagnosis was performed on each model. A generalized standard-error inflation factor was used to ensure absence of collinearity between independent variables. Linearity of the quantitative independent variables was checked through partial regression plots; goodness of fit was guaranteed by Hosmer-Lemeshow test. A 5% significance level was used to establish statistical significance. Specificity and sensitivity were assessed by using a 2 x 2 contingency table, and the level of agreement between the different tools was estimated using kappa statistics. Statistical data analysis was developed using the SPSS (SPSS, Chicago, IL) and R language software packages.

## Results

A total of 1,615 prescriptions made to 225 patients were reviewed. The mean age was 73.1 years (standard deviation 5.8), and 56.9% of patients were female. Most patients lived with their partner (59%) or family (16.5%), whereas 23% lived alone. The average CCI was 1.73, and 23.1% of patients had CCI scores >2. The most common diagnoses included hypertension (76.8%), bone and joint disorders (72.4%) and dyslipidemia (52.4%). Thirty-six per cent had diabetes mellitus and 46% suffered from insomnia. Ninety-five percent of outpatients had a Katz of A-B; the mean score on the Lawton scale was 6.6 (standard deviation 1.9). In total, 75.8% of patients had normal values on the GDS. The main characteristics of the study population are presented in Table 1.

**Table 1:** Characteristics of Study Population (n = 225).

Variable	Value
Age, mean ± SD	73.1 (±5.8)
Sex (Female), n (%)	128 (56.9)
Living alone, n (%)	51 (22.7)
Body Mass Index, mean ± SD	29.93 (±5.2)
Body Mass Index, n (%):	
Normal weight	26 (11.2)
Overweight	100 (44.8)
Obesity	59 (26.5)
Morbid obesity	38 (17)
Charlson Comorbidity Index, mean ± SD	1.73 (±1.8)
Charlson Comorbidity Index, n (%):	
0	66 (29.3)
1–2	107 (47.6)
3–4	30 (13.3)
5 or more	22 (9.8)
Most prevalent chronic diseases, n (%):	
Hypertension	172 (76.8)
Osteoarticular disease	163 (72.4)
Dyslipidemia	118 (52.4)
Insomnia	105 (46.7)
Peripheral vascular disease	97 (43.1)
Gastrointestinal disease	93 (41.3)
Diabetes mellitus	82 (36.4)
Psychopathology	77 (34.2)
Heart disease	72 (32.1)
SPMSQ (0–2 errors), n (%)	207 (92.8)
ADL (Katz: A-B), n (%)	209 (94.5)
IADL (Lawton: ≥6), n (%)	176 (78.5)
GDS-15, n (%):	
0–5	169 (75.8)
6–9	45 (20.2)
10 or more	9 (4)
Number of medications per patient, mean ± SD	7.1 (±3.8)
Medications (receipt of >1), n (%):	
Cardiovascular (C Group)	187 (83.1)
Digestive and Metabolism (A Group)	170 (75.5)
Nervous System (N Group)	159 (70.6)
Blood & blood forming organs (B Group)	106 (47.1)

SPMSQ: Short Portable Mental Status Questionnaire; ADL: Activity of Daily Living; IADL: Instrumental Activities of Daily Living; GDS-15: Geriatric Depression Scale (<5: no depression; >5: suggestive of depression; >10: almost always depression).

The median number of medications per patient was 7.1 drugs (standard deviation 3.8, range 0–19). The prevalence of elderly people exposed to polypharmacy (5 or more medications) was 72.9%, whereas 28.4% of the participants took ≥10 drugs regularly. The most widely prescribed ATC groups were C (cardiovascular, 83.1% of the patients took at least one drug of this group), A (alimentary tract and metabolism, 75.5%), N (nervous system, 70.6%) and B (blood and blood-forming organs, 47.1%). Omeprazole and paracetamol were the two most frequently used drugs, followed by aspirin, simvastatin, enalapril and metformin.

### Potentially Inappropriate Medications (PIMs)

PIMs were detected in 18.7% and 37.3% of patients according to the STOPP v1 and the 2012 AGS Beers criteria, respectively. This percentage rose to 40.4% when STOPP v2 (Table 2) was used. According to STOPP v1, the most common PIM were long-term long-acting benzodiazepines (31.42%). As to the 2012 AGS Beers criteria, PIMs were also prevailingly found in the central nervous system, more specifically, short-intermediate and long-acting benzodiazepines (52.4%), followed by dugs related to falls or fractures (10.5%). The most prevalent PIMs as detected with the updated version of STOPP were benzodiazepines prescribed for ≥ 4 weeks (38.6%), followed by drugs prescribed beyond the duration recommended (13.6%) and duplicate drug class prescriptions (7.6%).

**Table 2:** Sensitivity and Specificity of Each Tool.

CRITERIA					
	STOPP v1	2012 BC	STOPP v2	START v1	START v2
No. of PIMs or PPOs	42	84	91	78	49
PIM/PPO Prevalence (%)	18.7	37.3	40.4	34.7	21.8
(95% CI)	(14.1–24.2)	(31.3–43.8)	(34.2–46.9)	(28.7–41.1)	(16.8–27.6)
Sensitivity	Reference*	73.8	88.1	Reference*	52.6
(95% CI)	-	(58.8–84.8)	(74.5–95.3)	-	(41.6–63.3)
Specificity	Reference	71	70.5	Reference	94.6
(95% CI)	-	(64.1–77.1)	(63.5–76.6)	-	(89.5, 97.4)
Kappa statistic	Reference	0.32	0.40	Reference	0.52
(95% CI)	-	(0.20–0.44)	(0.29–0.51)	-	(0.40–0.64)

STOPP: Screening Tool of Older Person’s Potentially Inappropriate Prescriptions; 2012 BC: 2012 American Geriatrics Society Beers Criteria; START: Screening Tool to Alert doctors to the Right Treatment

* Chosen as the reference because it has been the first version of STOPP/START and for being the tool most widely used in Europe.

The prevalence rates for PIMs, as well as the sensitivity and specificity of each screening tool are displayed in Table 2. Taking the first version of STOPP as the reference point, AGS Beers criteria had a sensitivity of 73.8% and a specificity of 71% to detect PIMs. In the case of STOPP v2, its sensitivity to detect PIMs reached 88.1%, whereas its specificity was 70.5%. The level of agreement or Kappa index between the 2012 AGS Beers and the STOPP v1 was 0.32 (p<0.001), and 0.40 (p<0.001) for the two versions of STOPP. When crossing STOPP v2 with the 2012 Beers criteria, we obtained a kappa index of 0.47 (p<0.001).

According to the logistic regression model for the STOPP v2, the risk of PIM would be 14% greater for each drug added (OR: 1.14, 95% CI = 1.06–1.25). The presence of a psychological disorder (OR: 2.22, 95% CI = 1.13–4.37) and insomnia (OR: 3.35, 95% CI = 1.80–6.32) were found to be predictors of PIM use. There was no statistically significant association between sex or age and PIMs (Table 3).

**Table 3:** Multivariate Logistic Regression for Having at Least One PIM According to STOPP v2.

STOPP v2	
Variable	OR (95% CI)
*Age*	1.05 (0.99–1.10)
*No*. *of medications*	1.14 (1.06–1.25)*
*Sex*:	
Man	0.85 (0.45–1.62)
Woman	1
*Psychopathology*:	
One or more	2.22 (1.13–4.37)*
No	1
*Insomnia*:	
Yes	3.35 (1.80–6.32)^†^
No	1

* p<0.01;

^†^ p<0.001

STOPP v2: Screening Tool of Older Person’s Potentially Inappropriate Prescriptions version 2; OR (95% CI): Odds ratio (95% confidence interval).

### Potential Prescribing Omissions (PPOs)

START v1 identified a total of 113 PPOs in 78 patients (34.7%) (Table 2). In contrast, the prevalence of PPOs dropped to 21.8% (49 patients) after 61 potential omissions were detected with the updated version. According to START v1, the most frequent PPO was antiplatelet therapy in diabetes mellitus with major cardiovascular risk factors (24.8%), followed by statins in the same clinical situation (21.2%) and metformin with type 2 diabetes (14.1%). Thus, the endocrine system accounted for most omissions (62.8%). Conversely, START v2 indicated that most omissions occurred in the gastrointestinal system (29.5%), as the main PPO was fibre supplementation for diverticulosis. Other prevalent PPOs were bone anti-resorptive or anabolic therapy in osteoporosis (13.1%) and the use of angiotensin converting enzyme (ACE) inhibitor with systolic heart failure (11.4%). As a whole, the cardiovascular system accounted for 26.2% of the PPOs detected with START v2, whereas the endocrine system only accounted for 1.6%.

Using START v1 as the reference point, START v2 had a sensitivity of 52.6% and a specificity of 94.6% to detect PPOs. The Kappa index between the two versions of START was 0.52 (p<0.001) (Table 2).

After adjustment for functional status, age, sex, number of drugs taken and other prevalent diseases, only heart disease was found to be related to PPOs, according to the START v2 (OR: 2.5, 95% CI = 1.16–5.81).

## Discussion

The prevalence of PIMs changed according to the tool used. With STOPP v1, 18.7% of patients were found to receive potentially inappropriate prescriptions. Although numerous studies have used this tool in the last years, we only focused on comparisons of interest among community-dwelling patients. The figures obtained for Málaga–albeit close to other results [15, 16]–are below those reported in similar European studies [2, 4, 10, 17–19]. Differences in study design and prescription habits may explain the high variability observed in prevalence rates. Despite the conventional problems of applicability of Beers criteria in Europe, the updated version of 2012 has been proven to be superior to STOPP by a study conducted in Spain [10], which has been reproduced by other studies [11, 20]. Consistently, the results obtained in our study show that the 2012 AGS Beers criteria are more effective in detecting PIMs than the original STOPP tool (37.3% vs 18.7%). Nevertheless, the most interesting finding of our study was that the sensitivity to detect PIMs has been significantly improved in the updated version of STOPP, making it the most sensitive tool. Such improvement may be related to the increase in the number of criteria included in the updated version, namely antiplatelet/anticoagulant drugs, drugs affecting or affected by renal function and drugs that increase anticholinergic burden [13].

Consistently with most studies [14], this study revealed that the most common PIMs were benzodiazepines, which is one of the indicators that have been modified (benzodiazepines for ≥4 weeks). A contributing factor is the widespread use of this drug group in Spain, which is less frequent in other European countries [21]. In contrast, other PIMs traditionally reported in the literature, such as overdosing of aspirin and long-term use of NSAIDs (1.5% of PIMs in both cases), were scarcely detected in this study. The decrease in the prevalence of long-term use of NSAIDs may be due to recent notices published on its cardiovascular safety. This fact also evidences that the pattern of PIMs evolves over time, as shown in studies on trends in prescribing habits [22].

The level of agreement between the two versions of STOPP was found to be moderate, which can be explained by the significant changes made to the updated version, were new categories, newly approved drugs, new indicators based on updated evidence, modified indicators and new implicit criteria have been included, and other existing criteria have been removed due to weak or equivocal supporting evidence. The number of drugs taken was observed to be a risk factor for PIM, which is consistent with most previous studies [2, 10, 14, 15, 17, 19, 23]. Other predictors of PIM included a diagnosis of some psychopathology and insomnia. Of note, this is the first study to report insomnia as a predictor of PIM, since it is strongly related to the use of benzodiazepines.

A study performed in Turkey was recently published in which a similar PIM prevalence (39.9%) according to STOPP v2 was achieved, although with a different profile from that of ours. The most common criterion in this study was the use of >160 mg aspirin, followed by the use of loop diuretics in urinary incontinence [24].

The prevalence of PPOs detected using the first version of START (34.7%) was moderate as compared to other studies performed in Europe in the primary care setting, were percentages ranged between 22.7% and 50% [15–17, 19, 25, 26]. The highest rate of underprescription of beneficial agents was observed in the endocrine system, especially in patients with diabetes, which is in agreement with recent studies [17, 19, 26, 27]. The most significant finding was that the rate of omissions according to START v2 dropped 37% despite the increase in the number of items. This fact may be strongly associated with the withdrawal of aspirin and statin therapy for primary prevention of cardiovascular disease in diabetes mellitus, which were the most prevalent items in our sample according to version 1. Heart disease was also found to be a predictor of PPOs, which may be due to the high number of items related to cardiovascular factors and the prevalence of this type of disease in the population. Failure to comply with START-based recommendations may be associated with a high risk of severe ADEs, the use of other effective treatments and severe disability [28]. Nevertheless, identifying the reasons for inappropriate prescribing was not among the purposes of this study.

In our opinion, although physician’s implicit judgment and the individualization of treatments are necessary–especially in patients who are more difficult to manage- the STOPP/START criteria are a useful tool. Indeed, it is known that treatment appropriateness can be improved by 39% when these criteria are used in routine clinical practice [29].

A limitation of this study is its lack of external validity. Nevertheless, the sample of this study is representative of the population of older adults in the ambulatory setting. In contrast, a strength of this study is that it provides comprehensive direct data that improve the applicability of STOPP/START criteria and facilitates the estimation of PIMs and PPOs [30]. This study also contributes invaluable data on the most prevalent types of PIMs and identifies the predictors of treatment inappropriateness, which will serve to guide the development of interventions for improving prescription quality.

## Conclusions

The updated version of STOPP criteria is more sensitive than the first version and the 2012 AGS Beers criteria to detect PIMs. On the other hand, the updated version of START is less sensitive than START v1 to detect PPOs, and shows a clearly different profile of omissions. This fact evidences the significant modifications made to STOPP/START criteria based on new evidence and medications emerged since the development of the original criteria.
